# Efficacy of EGFR-TKIs with or without angiogenesis inhibitors in advanced non-small-cell lung cancer: A systematic review and meta-analysis

**DOI:** 10.7150/jca.34957

**Published:** 2020-01-01

**Authors:** Zhaoxin Chen, Jia Wei, Xiaoting Ma, Jing Yu

**Affiliations:** Cancer Center, Beijing Friendship Hospital, Capital Medical University, No. 95 Yong An Road, Xi Cheng District, Beijing, 100050, China.

**Keywords:** angiogenesis, NSCLC, EGFR-TKI

## Abstract

In the present study, we evaluated the efficacy and safety of epidermal growth factor receptor tyrosine kinases (EGFR-TKIs) combined with or without angiogenesis inhibitors in advanced non-small-cell lung cancer (NSCLC). We searched published randomized controlled trials (RCTs) comparing EGFR-TKIs with and without angiogenesis inhibitors for the treatment of advanced NSCLC. PubMed, EMBASE, PMC, the American Society of Clinical Oncology (ASCO) and the European Society of Medical Oncology (ESMO) databases were searched. The extracted data on progression-free survival (PFS) and overall survival (OS) were measured in terms of hazard ratios (HRs) and corresponding 95% confidence intervals (CIs). In addition, odds ratios (ORs) and corresponding 95% CIs were pooled for objective response rate (ORR) and disease control rate (DCR). Risk ratios (RRs) and corresponding 95% CIs were pooled for risk of adverse events (AEs). EGFR-TKIs combined with angiogenesis inhibitors showed significant improvements in PFS (HR 0.72, 95% CI 0.61-0.84, *P* <0.0001), ORR (OR 1.51, 95% CI 1.17-1.97, *P*=0.002) and DCR (OR 1.49, 95% CI 1.24-1.81, *P*<0.0001) compared with EGFR-TKIs combined with placebo. However, EGFR-TKIs combined with angiogenesis inhibitors failed to improve OS (HR 0.94, 95% CI 0.84-1.05,* P* = 0.26). In addition, diarrhea, hypertension, thrombocytopenia, neutropenia, fatigue, rash, and dermatitis acneiform were significantly increased in patients treated with angiogenesis inhibitors. Thus, EGFR-TKIs combined with angiogenesis inhibitors were superior to EGFR-TKIs alone in advanced NSCLC due to their effects on PFS, ORR and DCR, but the increased incidence of AEs had an influence on the tolerability of this combination therapy.

## Background

Lung cancer is the most common incident cancer and the leading cause of cancer death worldwide^1^. Non-small-cell lung cancer (NSCLC), which accounts for approximately 80% of all lung cancers, has a poor survival rate as it is at an advanced stage when diagnosed in the majority of cases^2^. The 5-year overall survival (OS) rate for NSCLC is less than 40%^3^. A clinically significant proportion of patients have activating mutations of the epidermal growth factor receptor (EGFR)^4^. In these patients, monotherapy with first or second generation EGFR-tyrosine kinase inhibitors (EGFR-TKIs) as first-line treatment for EGFR-mutated NSCLC is the standard therapy, and the median progression-free survival (PFS) with this treatment ranges from 9.2-14.7 months^5^-^7^. However, patients treated with EGFR-TKIs are likely to acquire resistance and cancer recurrence occurs within 1 year of treatment initiation^8^-^9^. To improve outcomes, synergistic combinations may be added to the initial treatment of EGFR-TKIs. Preclinical studies^10^-^11^ have shown promising results with the combination of anti-EGFR and anti-angiogenesis drugs in NSCLC. A clinical study^12^ compared erlotinib combined with or without sunitinib in 960 patients with refractory advanced NSCLC. The results showed that the median PFS was 3.6 months in the erlotinib plus sunitinib group versus 2.0 months in the erlotinib alone group (*P* = 0.0023), and the objective response rate (ORR) was 10.6% versus 6.9% (*P* = 0.0471), respectively. In contrast, the study by Spigel D R^13^ showed that the OS, PFS, ORR, and DCR were not different in patients with refractory advanced NSCLC administered erlotinib with or without sorafenib. Thus, the overall efficacy and safety of this combination in NSCLC are still confused. In this study, we performed a meta-analysis to update and summarize the efficacy and safety of EGFR-TKIs combined with angiogenesis inhibitors versus EGFR-TKIs combined with placebo in patients with advanced NSCLC.

## Methods

### Search strategy

**Figure 1:**
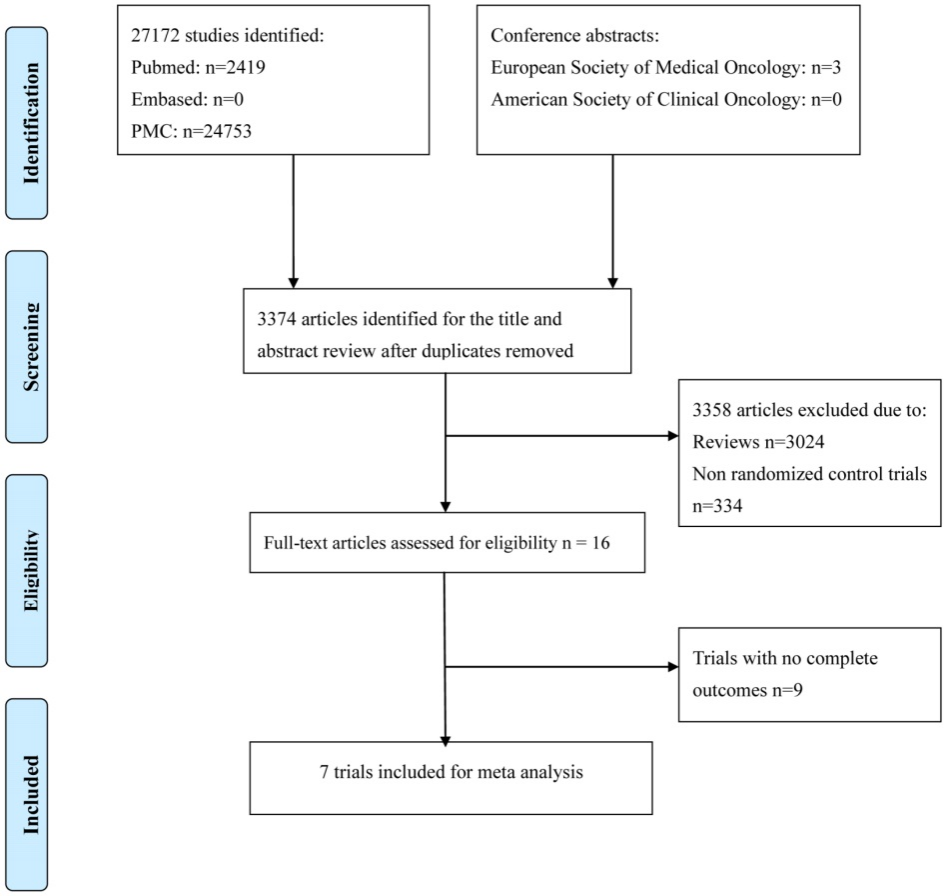
Flow chart of search process.

An electronic search of the PubMed, PMC and EMBASE databases as well as the American Society of Clinical Oncology (ASCO) and the European Society of Medical Oncology (ESMO) databases was performed from inception to March 2019. The detailed search strategy is described in Fig. 1. The search strategy included a combination of the MeSH term “angiogenesis inhibitors” or the keywords “angiogenetic inhibitors,” “angiogenic antagonists,” “angiogenic inhibitors,” “angiostatic agents,” “antiangiogenetic agents,” “angiogenesis factor inhibitor”; the MeSH term “epidermal growth factor receptor tyrosine kinase inhibitors” or the keywords “epidermal growth factor receptor inhibitors”; the MeSH term “non-small-cell lung cancer” or the keywords “lung cancer”. All potentially relevant studies were retrieved, and their references were checked for additional eligible studies.

### Definition of EGFR-TKIs and angiogenesis inhibitors

We defined angiogenesis inhibitors as drugs which targeted vascular epidermal growth factor (VEGF) and its receptors, which are the key mediators of angiogenesis, and EGFR-TKIs as drugs directed against epidermal growth factor receptor tyrosine kinase.

### Inclusion criteria

Studies which met the following criteria were included: (1) patients must be cytologically or pathologically confirmed as having NSCLC at a clinically advanced stage; (2) randomized controlled trials (RCTs) comparing EGFR-TKIs plus angiogenesis inhibitors with EGFR-TKIs plus placebo were eligible; (3) one or more of the following were reported in the trials: overall response rate (ORR) (the sum of complete response [CR] and partial response [PR]), disease control rate (DCR) (the sum of CR, PR and stable disease [SD]), PFS and OS.

### Data extraction

Two independent investigators extracted data from the included studies on the basis of the Preferred Reporting Items for Systematic Reviews and Meta-Analyses (PRISMA). When the two investigators disagreed, a third investigator participated in the discussion to resolve the disagreement. Information collected from these trials included the first author, year of publication, number of patients, median age, therapeutic regimen, doses, and outcomes. Clinical data collected from the trials included median PFS and median OS, hazard ratios (HRs) for OS and PFS and their 95% confidence intervals (CIs), DCR and ORR, odds ratios (ORs) for DCR and ORR, and their 95% CIs. The response was evaluated according to the Response Evaluation Criteria in Solid Tumors (RECIST, version 1.1) and classified as a CR, PR, SD, or progressive disease (PD). ORR was defined as CR with PR and DCR was defined as ORR with SD.

### Quality assessment

The risk of bias in each study was assessed using the Cochrane Collaboration tool. The following evaluation domains were assessed accordingly: randomization sequence generation, allocation concealment, blinding of participants and study personnel, blinding of outcome assessors, incomplete outcome data, selective reporting, and other biases. The risk of each domain was rated as high risk, unclear risk, or low risk according to the match level between information extracted and evaluation criteria.

### Statistical analysis

A statistical analysis was conducted, and forest plots were performed using Review Manager 5.3. ORs and their 95% CIs were calculated for DCR and ORR. HRs were summarized and their corresponding standard errors were computed to analyze the time-to-event data as generic inverse variance outcomes. The inverse variance algorithm and Mantel-Haenszel algorithm were used. Heterogeneity between studies was assessed with Cochrane's X^2^ statistics and the inconsistency statistic (I^2^). We considered I^2^ < 50% as low level heterogeneity and I^2^ > 50% as significant heterogeneity. A fixed-effect model was used when I^2^ < 50% and a random-effect model was used when I^2^ > 50%. *P* values < 0.05 were regarded as statistically significant in all included studies. Publication bias was evaluated according to the funnel plot and Begg's and Egger's tests using Review Manager 5.3.5.

## Results

### Characteristics of the included studies

**Figure 2:**
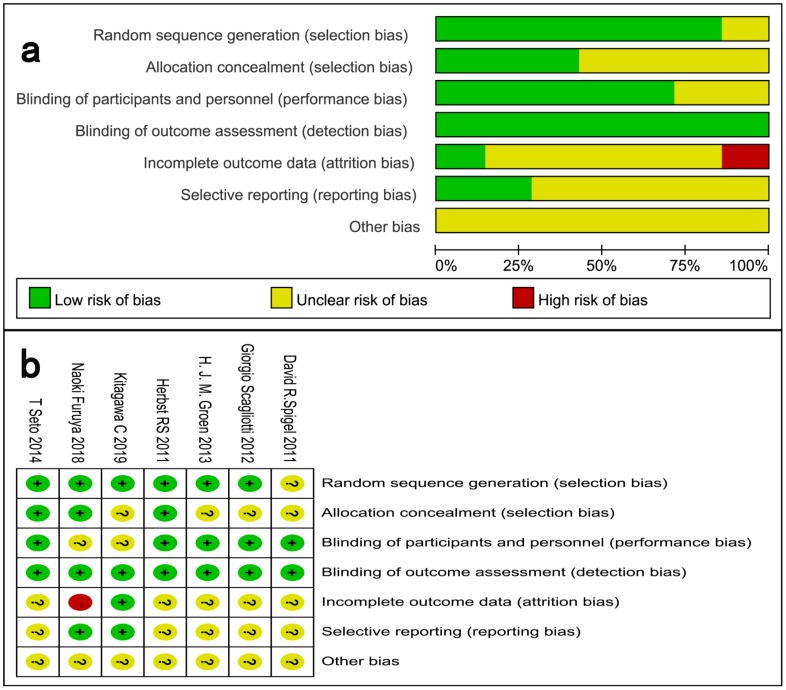
Assessment of risk of bias. (a) Risk of bias summary. (b) Risk of bias graph.

Figure 1 shows the flow chart of study selection. A total of 27172 relevant studies were identified following a comprehensive search, and 3 conference abstracts were obtained by manual searching of the ESMO database. 23801 articles were excluded as they were duplicates, leaving 3374 articles potentially eligible for inclusion, of which 3358 were eliminated after reading the titles and abstracts. The full texts of the remaining 16 articles were then reviewed, and seven trials^12^-^18^ involving 2285 patients were finally included in the meta-analysis. The sample size in the included trials varied from 15 to 960. Of these, four studies enrolled patients who were treated with bevacizumab, and three trials enrolled patients who were treated with multiple tyrosine kinase inhibitors. Four trials were conducted in the first-line setting and the other three trials in the second- or third-line setting. Table 1 and Fig. 2 summarize the characteristics and qualities of both the included agents and articles.

### Assessment of methodological quality

We critically assessed the methodological quality of the included studies in accordance with the Cochrane Collaboration Risk of Bias Tool. All included trials were rated as low bias risk during randomization, as the authors stated the principles of randomization in detail. Other bias sources were not identified. The graphical results of methodological quality are shown in Fig. 2. The risk of bias items for each included study are presented in Fig. 2.

### Overall survival (OS) and progression-free survival (PFS)

**Figure 3:**
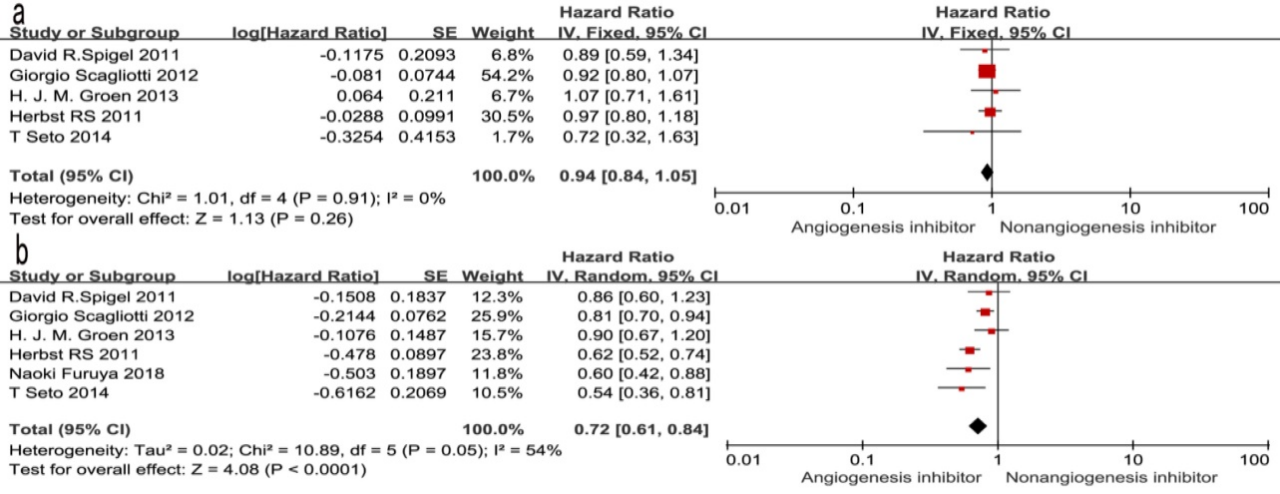
Forest plot and pooled HR and 95% CI for OS (a) and PFS (b): anti-angiogenesis therapy versus non-anti-angiogenesis therapy. Annotation: The pooled HR for OS and PFS showed that the patients receiving anti-angiogenesis therapy demonstrated a significant improvement in PFS. Abbreviations: HR, hazard ratios; OS, overall survival; PFS, progression-free survival; CI, confidence intervals.

Of the seven trials, all included studies reported PFS, and four trials reported OS. Four reported a statistically significant improvement in OS and six trials showed improved PFS. The median OS in the EGFR-TKIs plus angiogenesis inhibitor groups reported in four trials ranged from 7.23 to 9.3 months, and the median PFS ranged from 1.7 to 16.6 months. The pooled results showed that compared with the EGFR-TKIs alone groups, treatment with angiogenesis inhibitors was associated with a significantly prolonged PFS (HR 0.72, 95% CI 0.61-0.84, *P* < 0.0001, Fig. 3b). Significant heterogeneity was detected among the studies as shown in Fig. 3b (*P* =0.05, I^2^ = 54%); thus, we conducted a sensitivity analysis. We excluded the study by Giorgio Scagliotti that had the maximum relative weight (25.9%) and the study by T. Seto which had the minimum relative weight (10.5%) shown in Fig. 3b. The survival outcome was similar. In the subgroup analyses, PFS was significantly improved following treatment with both bevacizumab combined with EGFR-TKIs (HR 0.61, 95% CI 0.52-0.70, *P* < 0.00001, Fig. 3b) and multikinase inhibitors combined with EGFR-TKIs (HR 0.83, 95% CI 0.73-0.94, *P* = 0.003, Fig. 3b) compared to EGFR-TKIs alone. However, both bevacizumab and multikinase inhibitors were unable to prolong OS compared to EGFR-TKIs alone. With regard to the line of treatment, angiogenesis inhibitors failed to increase OS in the first-, second- or third-line treatments. Both first-line (HR 0.57, 95% CI 0.44-0.76, *P* < 0.0001) and second- or third-line (HR 0.75, 95% CI 0.68-0.83, *P* < 0.00001) treatments with angiogenesis inhibitors plus EGFR-TKIs increased PFS.

### Overall response rate (ORR) and disease control rate (DCR)

**Figure 6:**
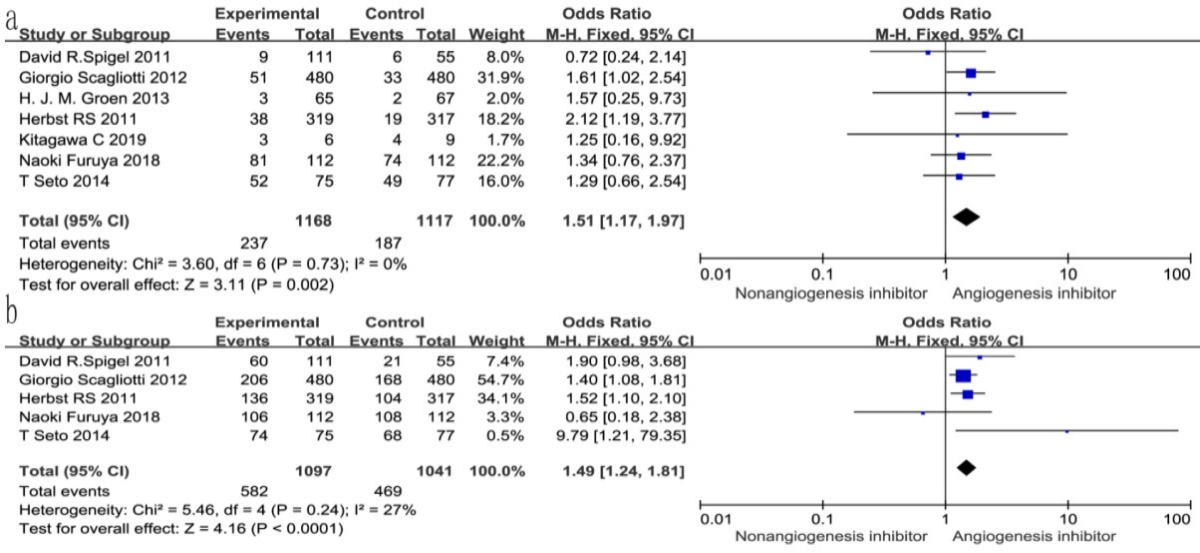
Forest plot and pooled RR and 95% CI for DCR (a) and ORR (b): anti-angiogenesis therapy versus non-anti-angiogenesis therapy.

**Figure 7:**
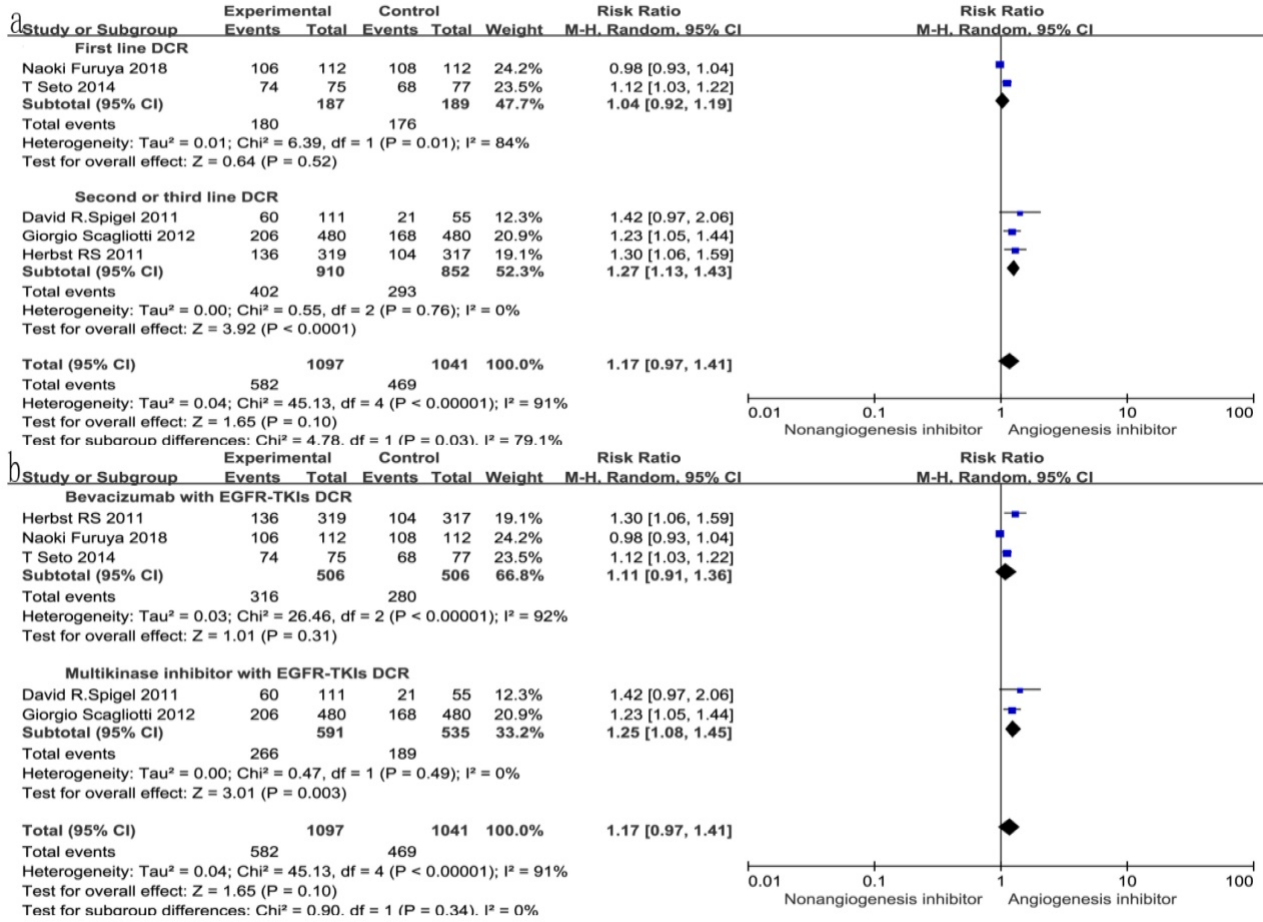
Forest plot and pooled RR and 95% CI for subgroup DCR: anti-angiogenesis therapy versus non-anti-angiogenesis therapy. Abbreviations: RR, risk ratios; CI, confidence intervals; DCR, disease control rate.

**Figure 8:**
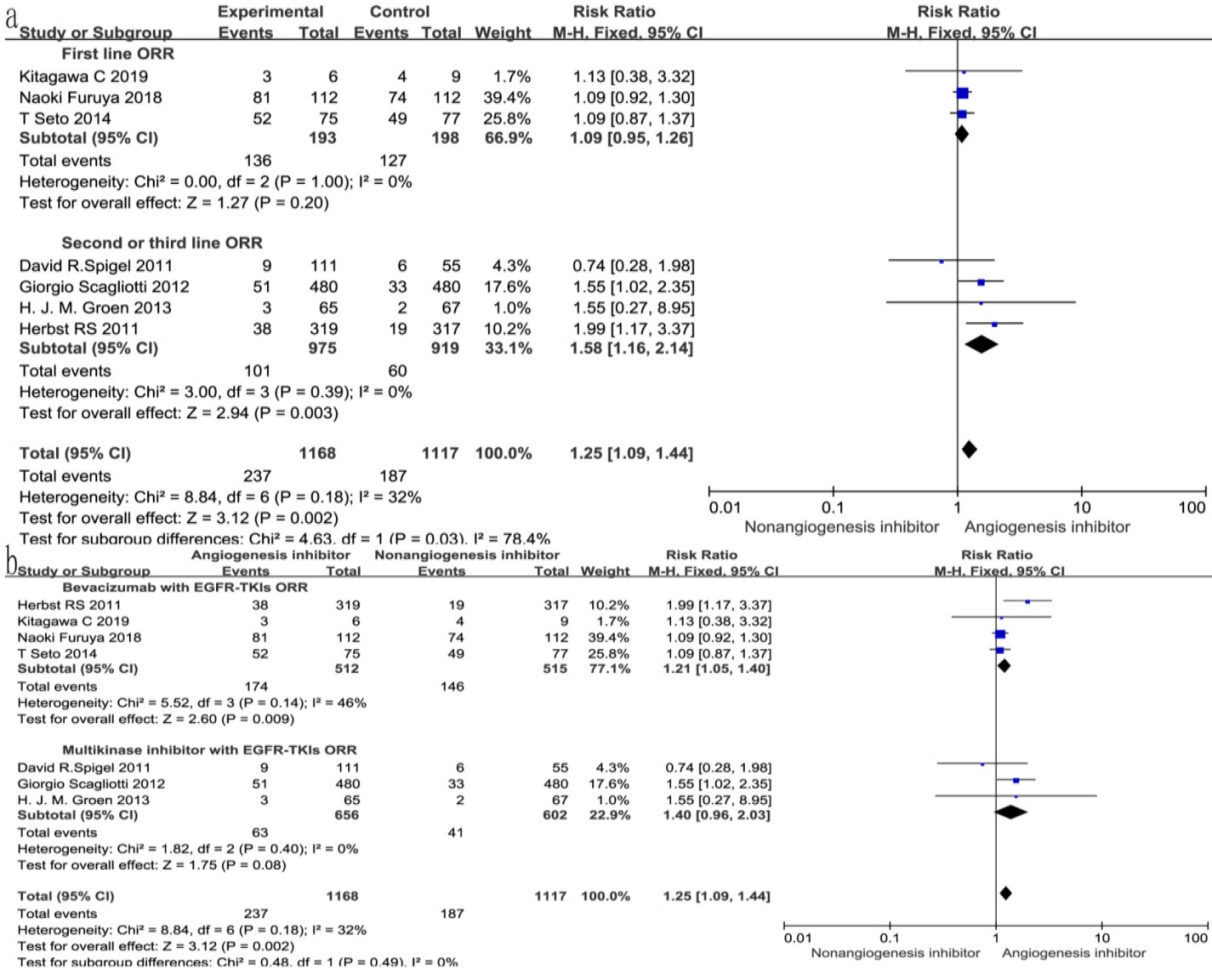
Forest plot and pooled RR and 95% CI for subgroup ORR: anti-angiogenesis therapy versus non-anti-angiogenesis therapy. Abbreviations: RR, risk ratios; CI, confidence intervals; ORR, overall response rate.

All seven trials reported ORR, and six studies reported DCR. The DCR ranged from 34 to 100%, and the ORR ranged from 4.6 to 72.3% in the EGFR-TKIs combined with angiogenesis inhibitors groups. The pooled data showed that angiogenesis inhibitors resulted in superior ORR (OR 1.52, 95% CI 1.17-1.97, *P* = 0.002, Fig. 6a) and DCR (OR 1.49, 95% CI 1.24-1.81, *P* < 0.0001, Fig. 6b) compared with non-angiogenesis inhibitors. Subgroup analysis of drug administration indicated that the multikinase inhibitors increased the DCR (RR 1.25, 95% CI 1.08-1.45, *P* = 0.003, Fig. 7b), while bevacizumab did not. On the contrary, bevacizumab increased the ORR (RR 1.21, 95% CI 1.05-1.40, *P* = 0.009, Fig. 8b), while multikinase inhibitors did not.

With regard to the line of treatment, angiogenesis inhibitors increased the DCR in second- or third-line treatment (RR 1.27, 95% CI 1.13-1.43, *P* < 0.0001, Fig. 7a), and there was a tendency to increase DCR in first-line treatment but there was no statistical significance (RR 1.27, 95% CI 1.13-1.43, *P* =0.52, Fig. 7a). ORR increased in second- and third-line treatments (RR 1.58, 95% CI 1.16-2.14, *P* = 0.003, Fig. 8a), but showing no statistical significance in first-line (RR 1.09, 95% CI 0.95-1.26, *P* = 0.2, Fig. 8a).

### Safety

Toxicities reported in the included studies are summarized according to the National Cancer Institute Common Toxicity Criteria in Table 2 (only grade ≥ 3 toxicities are presented). In general, grade ≥ 3 AEs were more frequent in patients treated with angiogenesis inhibitors and included hypertension (RR 6.41, 95% CI 3.77-10.91, *P*<0.00001), hemorrhage (RR 2.14, 95% CI 1.07-4.28, *P* = 0.03) and proteinuria (RR 15.18, 95% CI 2.02-113.88, *P*= 0.008) for anti-angiogenic-induced events, and neutropenia (RR 7.60, 95% CI 2.50-23.09, *P* = 0.0003), thrombocytepenia (RR 3.98, 95% CI 1.36-11.63, *P* = 0.01), diarrhea (RR 5.70, 95% CI 3.50-9.30, *P*< 0.00001) and fatigue (RR 2.21, 95% CI 1.40-3.51, *P* = 0.0007) for EGFR-TKIs induced events. In addition, decreased appetite (RR 3.43, 95% CI 1.62-7.26, *P* = 0.001) and dysgeusia (RR 3.22, 95% CI 1.48-7.02, *P* = 0.003) were significantly increased in patients treated with angiogenesis inhibitors and EGFR-TKIs. However, anemia (RR 1.32, 95% CI 0.76-2.31, *P* = 0.32), vomiting (RR 1.90, 95% CI 0.61-5.93, *P* = 0.27), rash (RR 1.23, 95% CI 0.69-2.22, *P* = 0.48) , thrombosis(RR 1.02, 95% CI 0.26-3.99, *P* = 0.98), interstitial lung disease(RR 0.72, 95% CI 0.14-3.64, *P* = 0.69) and nausea (RR 3.31, 95% CI 1.01-10.88, *P* = 0.05) in both groups of patients were not significantly different (*P* > 0.05). The RRs of grade ≥ 3 AEs are summarized in Table 2.

## Discussion

Lung cancer is the leading cause of cancer-related death worldwide, and most patients with NSCLC have advanced or metastatic disease at diagnosis. Erlotinib and gefitinib are oral EGFR-TKIs and have been proved to have superior effects in prolonging OS and PFS, especially in patients with EGFR mutations. However, most patients treated with EGFR-TKIs developed acquired resistance^19^-^21^. Tumor vessel abnormality and heterogeneity hinder drug delivery and effective cancer therapy. As shown in previous studies^10^,^22^, angiogenesis inhibitors normalized tumor vasculature which improved tumor perfusion, uptake of anticancer drugs and the efficacy of chemotherapy in neuroblastoma. Preclinical studies^23^-^24^ have revealed that acquired EGFR-TKIs resistance was significantly associated with dose. The higher the dose, the lower the incidence of EGFR-TKIs resistance. Furthermore, it was reported that combined VEGFR/EGFR pathway blockade abrogated primary or acquired resistance to EGFR inhibitors in four resistant NSCLC cell models^10^. Thus, EGFR-TKIs plus angiogenesis inhibitors may delay the appearance of EGFR-TKIs resistance by maintaining a higher dose of EGFR-TKIs. In conclusion, targeting the VEGF and the EGFR signaling pathways may resolve the problem of acquired resistance to EGFR inhibitors^25^. However, there is still controversy regarding the effects of EGFR-TKIs combined with angiogenesis inhibitors in advanced NSCLC. Facing up to this controversy, we performed this updated meta-analysis to summarize valuable information on the treatment of advanced NSCLC. Our results indicated that the combination of EGFR-TKIs and angiogenesis inhibitors resulted in substantial improvements in PFS, ORR and DCR compared with EGFR-TKIs combined with placebo, but had no effect on OS.

A retrospective study^26^ reported that an EGFR-TKI combined with bevacizumab achieved a median OS of 13.5 months and a median PFS of 4.1 months in 24 EGFR-mutant NSCLC patients who had acquired resistance to EGFR-TKIs, especially in T790M-negative patients. Herbst R S^16^ compared 319 patients treated with bevacizumab plus erlotinib and 317 patients treated with placebo plus erlotinib. No difference in median OS was observed between the two groups. In the study by Giorgio Scagliotti^12^, the median OS was 9.0 months in the bevacizumab plus erlotinib group and 8.5 months in the placebo plus erlotinib group, respectively (*P* = 0.1388). Furthermore, the median PFS was 3.0 months versus 2.6 months (*P* = 0.0023). Our results indicated that PFS improved in the EGFR-TKIs plus angiogenesis inhibitor groups; however, there was no OS benefit. This was consistent with the aforementioned data. In the study designed by Broglio K R^27^, the researchers divided OS into PFS and survival post-progression (SPP). The study showed that the probability of detecting a statistically significant difference in OS decreased dramatically when median SPP was more than 2 months. In addition, the study suggested that the longer the SPP, the less well the PFS‐HR reflected the OS‐HR. In this meta-analysis, only 4 of the included 7 RCTs provided a specific median OS and median PFS (in months). Median SPPs were more than 2 months in all four studies. For example, the least median SPP was 4.24 months in the study designed by David R. Spigel^13^. However, lack of statistical significance in OS could not fully imply the real benefit in OS, especially in this meta-analysis with a long median SPP. On the other hand, the stabilization or even shrinkage of tumor magnitude did affect the PFS but did not always affect the OS, especially in patients with a smaller tumor burden. It has been shown that, for this type of patient, even if the tumor was reduced and achieved SD, the benefit of PFS could not be converted into OS benefit^28^. Besides, even if the targeted therapy combinations achieved PFS benefit, they might change the biological characteristics of the tumor, which may undergo selective pressure, thus offsetting the treatment effect of the early stages and causing no improvement in OS^29^.

In all 7 RCTs included, the ORR ranged from 4.6% to 72.3% in the EGFR-TKIs plus angiogenesis inhibitors groups. With regard to the line of treatment, there was a tendency for increased ORR and DCR in first-line treatment but this tendency was not statistically significant. However, the ORR and DCR were significantly increased in second- and third-line treatments. These results could also be explained by the theory of selective pressure. As mentioned above, first-line treatment may change the biological characteristics of the tumor, which then undergoes selective pressure finally causing escape pathways for tumor^30^. In fact, a large number of mechanisms of acquired resistance have been discovered in tumor-cell clones and have evolved and proliferated under the selective pressure of initially effective treatment^31^. Under these circumstances, EGFR-TKIs combined with angiogenesis inhibitors could increase the ORR and DCR much better than EGFR-TKIs alone in second- and third-line treatment due to the blocking of multiple pathways and circumventing some mechanisms of resistance. EGFR-TKIs combined with bevacizumab can increase the ORR, while EGFR-TKIs combined with multikinase inhibitors tend to increase the ORR but the difference was not statistically significant (*P* =0.08). The EGFR-TKIs combined with multikinase inhibitors failed to increase the ORR. This may have been due to the small sample size of 1258 patients from only 3 RCTs. The DCR was increased following treatment with EGFR-TKIs combined with multikinase inhibitors, but not with EGFR-TKIs combined with bevacizumab. This might have been due to the multikinase inhibitors inhibiting not only VEGFR-1, -2, and -3, but also other signal pathways associated with proliferation, invasion and metastasis of the tumor such as platelet-derived growth factor receptor (PDGFR), stem-cell factor receptor (KIT), FMS-like tyrosine kinase 3 (FLT3), and colony-stimulating factor 1 receptor (CSF-1R), while bevacizumab inhibits only VEGF^32^-^34^.

In this meta-analysis, rash (16.6%), hypertension (14.7%) and diarrhea (13.3%) were the most common AEs in the combination group, while rash (11.5%), anemia (3.8%) and fatigue (3.4%) were common in the EGFR-TKI alone group. Diarrhea and skin toxicity are the most common AEs of EGFR-TKIs^35^, while hypertension, proteinuria, hemorrhage and arterial thromboembolic events are the most common AEs in patients treated with angiogenesis inhibitors. As previously mentioned, anemia, vomiting, rash, thrombosis, interstitial lung disease and nausea in both groups of patients were not significantly different (*P* > 0.05). However, grade ≥ 3 AEs were more frequent in patients treated with angiogenesis inhibitors and included hypertension, hemorrhage and proteinuria for anti-angiogenic-induced events, and neutropenia, thrombocytopenia, diarrhea and fatigue for EGFR-TKIs induced events. In addition, decreased appetite and dysgeusia were significantly increased in patients treated with EGFR-TKIs combined with angiogenesis inhibitors. Adverse events are one of the most relevant factors on the curative effect and survival. As previously mentioned, grade ≥ 3 AEs were more frequent in patients treated with angiogenesis inhibitors. Therefore, effective management of AEs is an important aspect of the overall treatment strategy for patients with advanced NSCLC, with the goal of maximizing therapy exposure, and thus achieving optimal clinical benefit.

There are many limitations in this meta-analysis. Firstly, only seven RCTs were included, and there were no subgroups related to ethnicity, EGFR mutation type or pathological classification. The influence of EGFR mutation or EGFR expression status could not be assessed. EGFR expression status was reported in a small number of patients. Secondly, the differences between statistical quality and follow-up time resulted in heterogeneity. Finally, this was a trial-level meta-analysis based on studies and not on individual patient data. Confounding variables such as patient co-morbidities, extent of disease, and differences in other possible prognostic factors could not be incorporated into this analysis. Therefore, future research should focus on high quality studies and clinical features in patients comprehensively evaluated, thus resulting in more standardized research and more accurate conclusions.

## Conclusions

EGFR-TKIs combined with anti-angiogenic treatment were better than EGFR-TKIs alone in terms of PFS, ORR and DCR in patients with advanced NSCLC. The benefit of combining EGFR-TKIs with angiogenesis inhibitors must be balanced by increased toxicity. Additional studies on this combination with respect to potentially predictive biomarkers are warranted.

## Figures and Tables

**Figure 4:**
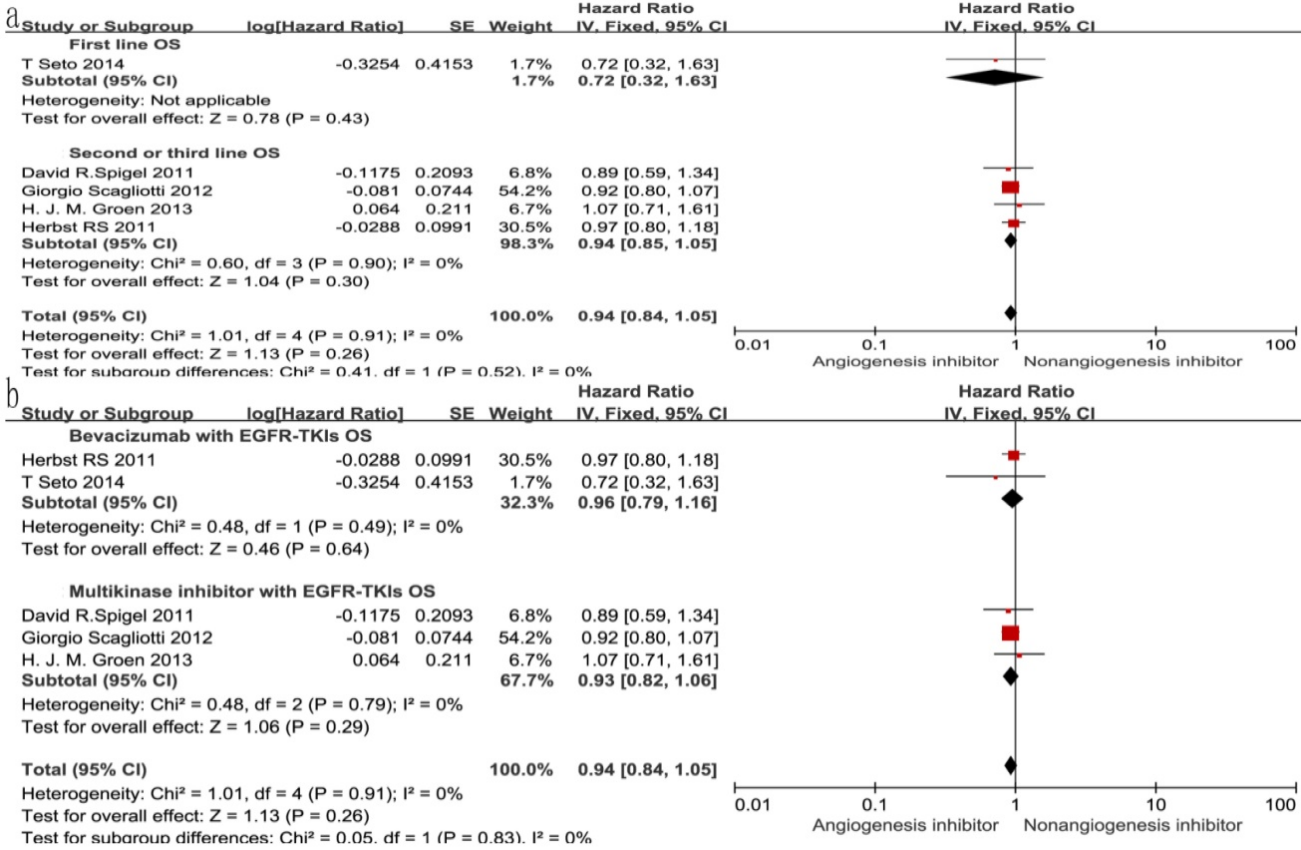
Forest plot and pooled HR and 95% CI for subgroup OS.

**Figure 5:**
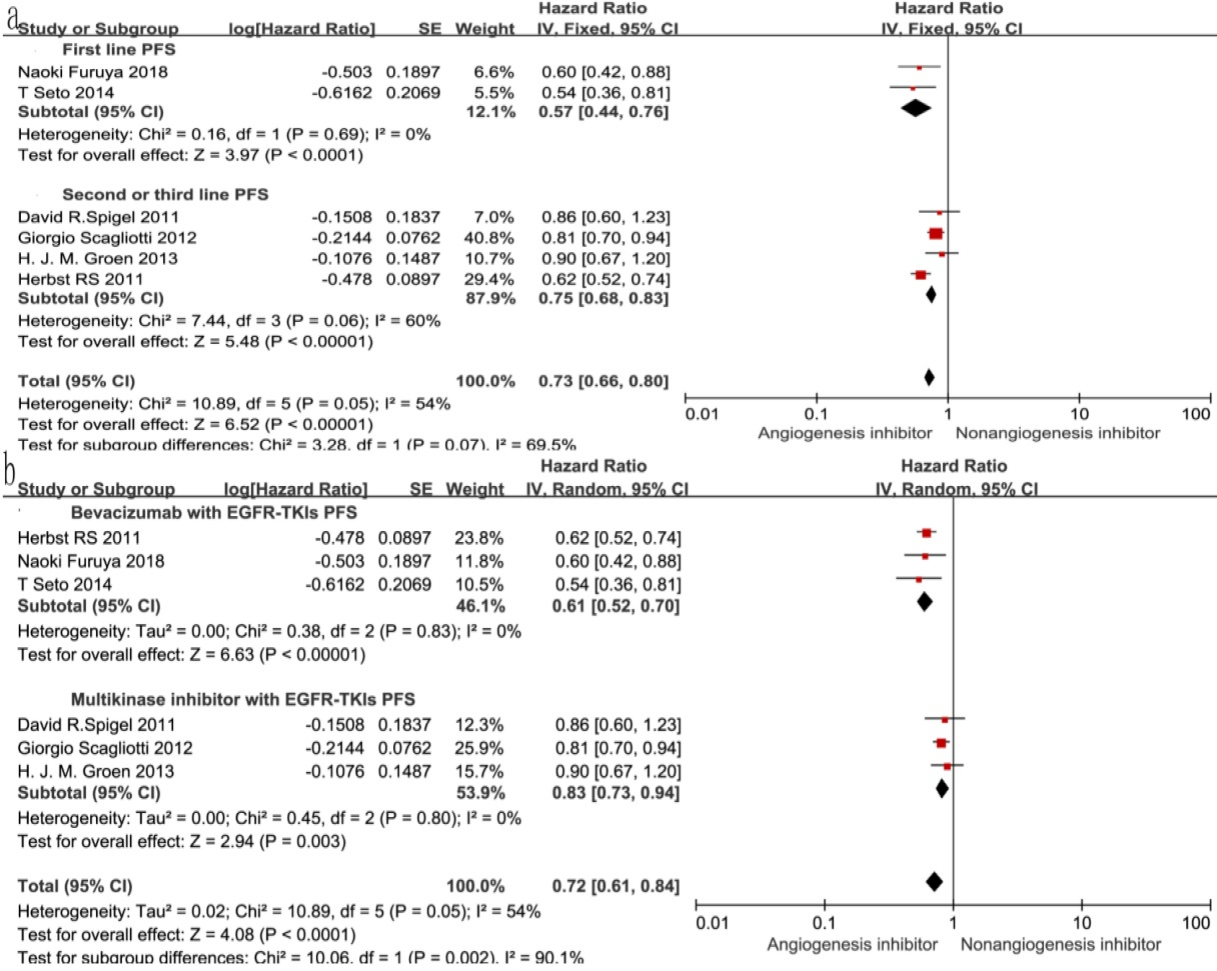
Forest plot and pooled HR and 95% CI for subgroup PFS.

**Table 1:** Characteristics of the included studies

Study/ Year	Country	Ethnicity	Line of treatment	Phase	Regimens	Number of patients	Median OS (months)	Median PFS (months)	ORR (%)	DCR (%)
David R. Spigel/ 2011	USA	Non-Asian	Second or third	II	Sorafenib + erlotinib vs. placebo + erlotinib	112 56	7.62 7.23 (HR 0.89, 95% CI 0.59-1.34, *P* = 0.290)	3.38 1.94 (HR 0.86, 95% CI 0.60-1.22, *P* = 0.196)	8.1 10.9 (*P* = 0.56)	54 38 (*P* = 0.056)
Giorgio Scagliotti/ 2012	Poland	Non-Asian Asian	Second or third	III	Sunitinib + erlotinib vs.placebo + erlotinib	480 480	9 8.5 (HR 0.922, 95% CI 0.797 - 1.067, *P* = 0.1388)	3.6 2 (HR 0.807, 95% CI 0.695-0.937, *P* = 0.0023)	10.6 6.9 (*P* = 0.0471)	42.9 35
H. J. M. Groen/ 2013	USA	Non-Asian Asian	Second or third	II	Sunitinib + erlotinib vs. placebo+erlotinib	65 67	8.2 7.6 (HR 1.066, 95% CI 0.705-1.612, *P* = 0.617)	2.8 2 (HR 0.898, 95% CI 0.671-1.203, *P* = 0.321)	4.6 3.0 (*P* = 0.624)	NR
Roy S Herbst/ 2011	USA	Non-Asian Asian	Second	III	Bevacizumab + erlotinib vs.erlotinib	319 317	9.3 9.2 (HR 0.97, 95% CI 0.80-1.18, *P* =0.7583)	3.4 1.7 (HR 0.62, 95% CI 0.52-0.75)	6 13	34 46
Takashi Seto/2014, 2018	Japan	Asian	First	II	Bevacizumab + erlotinib vs.erlotinib	75 77	(HR 0.73, 95% CI 0.32-1.63, *P* =0.7583)	16 9.7 (HR 0.54, 95% CI 0.36-0.79, *P* =0·0015)	69 63	98 88
Naoki Furuya/ 2018	USA	Non-Asian	First	III	Bevacizumab + erlotinib vs.erlotinib+ placebo	112 112	NR NR	16.9 13.3 (HR 0.605, 95% CI 0.417-0.877, *P* =0·01573)	72.3 66.1	94.6 96.4
Kitagawa C/ 2019	Japan	Asian	First	II	Bevacizumab+ gefitinib vs. gefitinib	6 9	NR NR	5.4 15.1	50 44	100 100

Abbreviations: OS, overall survival; PFS, progression-free survival; ORR, objective response rate; DCR, disease control rate

**Table 2:** RR of grade ≥ 3 adverse events in patients with advanced NSCLC treated with angiogenesis inhibitors

Grade≥3 adverse	No of trials	Events/total Treatment group	Control group	RR (95 % CI)	*P* value	Analysis model	
Decreased appetite	4	55/723	8/673	3.43 (1.62, 7.26)	0.001		Fixed
Vomiting	3	8/612	4/618	1.90(0.61,5.93)	0.27		Fixed
Anemia	3	30/584	20/532	1.32 (0.76, 2.31)	0.32		Fixed
Diarrhea	5	111/835	18/785	5.70(3.50, 9.30)	<0.00001		Fixed
Nausea	3	11/612	3/618	3.31 (1.01, 10.88)	0.05		Fixed
Hypertension	4	90/611	13/557	6.41 (3.77, 10.91)	<0.00001		Fixed
Hemorrhage	4	24/973	11/979	2.14(1.07, 4.28)	0.03		Fixed
Thrombocytopenia	3	16/648	3/596	3.98 (1.36, 11.63)	0.01		Fixed
Neutropenia	2	30/584	3/532	7.60 (2.50, 23.09)	0.0003		Fixed
Fatigue	4	60/723	23/673	2.21 (1.40,3.51)	0.0007		Fixed
Rash	7	191/1148	126/1098	1.23 (0.69,2.22)	0.48		Random
Dysgeusia	4	31/723	8/673	3.22(1.48,7.02)	0.003		Fixed
Proteinuria	3	14/187	0/189	15.18(2.02,113.88)	0.008		Fixed
Thrombosis	2	4/187	4/182	1.02(0.26,3.99)	0.98		Fixed
Interstitial lung disease	2	2/388	3/390	0.72(0.14,3.64)	0.69		Fixed
							

RR, risk ratios.
